# COVID-19 infection with complicated fulminant myocarditis: a case report

**DOI:** 10.1097/CP9.0000000000000050

**Published:** 2023-06-02

**Authors:** Kun Miao, Jinsheng Lai, Feng Wang, Luyun Wang, Chunxia Zhao, Dao Wen Wang

**Affiliations:** 1 Division of Cardiology, Department of Internal Medicine, Tongji Hospital, Tongji Medical College, Huazhong University of Science and Technology, Wuhan 430030, China.; 2 Hubei Key Laboratory of Genetics and Molecular Mechanisms of Cardiological Disorders, Huazhong University of Science and Technology, Wuhan 430030, China.

**Keywords:** COVID-19, Fulminant myocarditis, Myositis

## Abstract

Herein, we report the case of a young female patient who suffered from myositis and heart failure due to fulminant myocarditis induced by the 2019 coronavirus disease (COVID-19). After receiving intra-aortic balloon pump (IABP) and immunomodulatory treatment, her vital signs gradually stabilized and the IABP was removed. Cardiac and muscle magnetic resonance imaging confirmed extensive myocardial and skeletal muscle edema. Though it is not uncommon for COVID-19 infection to be complicated by myocarditis and myositis, such serious muscle injury warrants clinical vigilance.

## INTRODUCTION

Severe acute respiratory syndrome coronavirus 2 (SARS-CoV-2) infects host cells through the angiotensin-converting enzyme 2 (ACE2) receptor, the 2019 coronavirus disease (COVID-19) can lead to pneumonia with concurrent multiple organ and tissue injuries including to the myocardium, liver, and kidney^[1,2]^. With accumulated experience treating patients infected by COVID-19, medical workers have paid special attention to heart injury, and thus saved more patients. In addition to the myocardium, the skeletal muscles (i.e., the largest tissue involved in glucose metabolism^[3,4]^), seems to be among the tissues affected by COVID-19^[5,6]^. Muscle pain is a key symptom that develops during the first three days of infection in those hospitalized with COVID-19^[7–9]^. Herein, we report the case of a young female patient with myocarditis and muscle injury induced by COVID-19.

## CASE PRESENTATION

Written informed consent was obtained from the guardian of the patient for publication of this case report and accompanying images. A copy of the written informed consent is available for review by the editorial office of this journal.

A 36-year-old female developed fever with peak temperature of 38°C, accompanied by cough and expectoration, 1 week before admission at a community hospital. COVID-19 infection was confirmed by an in-hospital nucleic acid test. Two days later, her body temperature returned to normal, but she developed palpitations, chest tightness, and obvious general fatigue and muscle pain, predominantly in both lower limbs. There was no chest pain, syncope, abdominal pain, or diarrhea. No signs of viral pneumonia or pericardial effusion were found on lung computed tomography. Troponin, myoglobin, and creatine phosphokinase-MB(CK-MB) were increased (high-sensitivity cardiac troponin I [hs-cTnI] was 508.7 pg/mL, myoglobin was higher than 1,200.0 ng/mL, and CK-MB was 37.2 ng/mL). Considering COVID-19 infection complicated with myocarditis, the patient was referred to our hospital for further diagnosis and treatment. She did not report any history of cardiovascular disease or diabetes. She had received the COVID-19 vaccination during 2020–2021, with her most recent vaccination administered in November 2021.

Physical examination revealed a body temperature of 36.4°C, blood pressure of 96/70 mmHg (maintained with dopamine of 5 µg/kg/min), heart rate of 120 bpm, and respiratory rate of 30 breaths/min. Breathing sounds in both lungs were clear and no obvious dry or moist rales were heard. Sinus tachycardia, dull heart sound, uniform heart rhythm, and no obvious murmur were all recorded. There was no tenderness in the abdomen and no edema in both lower limbs.

**Figure 1.**
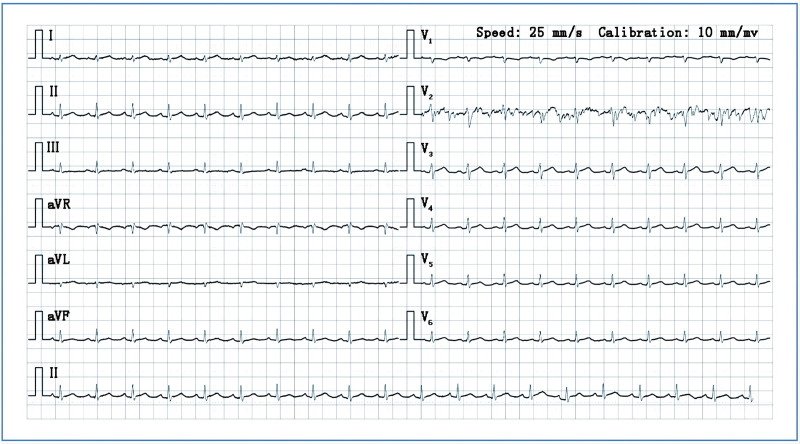
**12-Lead electrocardiogram**. Electrocardiogram after admission showed sinus tachycardia (heart rate 120bpm) and low voltage in limb leads. aVF: the augmented unipolar left leg lead; aVL: the augmented unipolar left arm lead; aVR: the augmented unipolar right arm lead.

Biochemical analyses were performed at admission (Table 1). Electrocardiography revealed sinus tachycardia and low voltage in the limb leads (Figure 1). Echocardiography showed left ventricular edema and systolic dysfunction (left ventricle 3.9 cm, left atrium 2.3 cm, interventricular septum 1.3 cm, posterior wall 1.3 cm, ejection fraction 46%, global longitudinal strain −5.9%), weakened left ventricular inferior wall motion, and moderate pericardial effusion. There was no obvious abnormality in the arteriovenous ultrasound of either lower limb.

**Table 1:** Clinical laboratory results

Item	1 day after admission	4 days after admission	10 days after admission	1-month follow-up	Reference range
White cell count, 10^9^/L	12.4	14.1	11.9	8.4	3.5–9.5
Red cell count, 10^12^/L	5.9	4.0	3.5	4.3	3.8–5.1
Absolute neutrophil count, 10^9^/L	10.7	11.4	9.1	6.2	1.8–6.3
Absolute lymphocyte count, 10^9^/L	1.1	1.4	1.9	1.4	1.1–3.2
Platelet count, 10^9^/L	140.0	109.0	276.0	246.0	125.0–350.0
Hemoglobin, g/L	174.0	119.0	108.0	132.0	115.0–150.0
Hematocrit, %	50.4	38.4	34.0	40.3	35.0–45.0
Bicarbonate radical, mmol/L	16.7	26.4	22.5	26.2	22.0–29.0
Glucose, mmol/L	8.8	–	–	–	3.9–6.1
Blood urea nitrogen, mmol/L	4.1	6.4	6.5	3.1	2.6–7.5
Creatinine, µmol/L	52.0	57.0	55.0	60.0	45.0–84.0
Total protein, g/L	53.9	48.4	63.7	–	64.0–83.0
Albumin, g/L	32.2	28.5	40.5	–	35.0–52.0
Total bilirubin, µmol/L	5.1	10.5	5.7	–	≤21.0
Procalcitonin, ng/mL	<0.05	–	–	–	0.02–0.05
Alanine aminotransferase, U/L	26.0	176.0	82.0	10.0	≤33.0
Aspartate aminotransferase, U/L	62.0	249.0	32.0	24.0	≤32.0
Alkaline phosphatase, U/L	52.0	45.0	64.0	–	35.0–105.0
Lactate dehydrogenase, g/L	448.0	616.0	311.0	–	135.0–214.0
Creatine kinase, U/L	3,296.0	10,455.0	372.0	111.0	≤190.0
Venous lactate, mmol/L	6.0	–	–	–	0.5–2.2
Interleukin 1β, pg/mL	<5.0	–	–	<5.0	<5.0
Interleukin 2 receptor, U/mL	362.0	–	–	152	223.0–710.0
Interleukin 6, pg/mL	4.2	–	–	1.8	<7.0
Interleukin 8, pg/mL	19.8	–	–	<5.0	<62.0
Interleukin 10, pg/mL	20.0	–	–	<5.0	<9.1
Tumor necrosis factor α, pg/mL	6.8	–	–	<4.0	<8.1
cTNI, pg/mL	380.7	175.4	–	<1.9	≤15.6
NT-proBNP, pg/mL	7,421.0	745.0	–	17.0	<116.0

“–” means no data.

cTNI: cardiac troponin I; NT-proBNP: N-terminal pro B-type natriuretic peptide.

**Figure 2.**
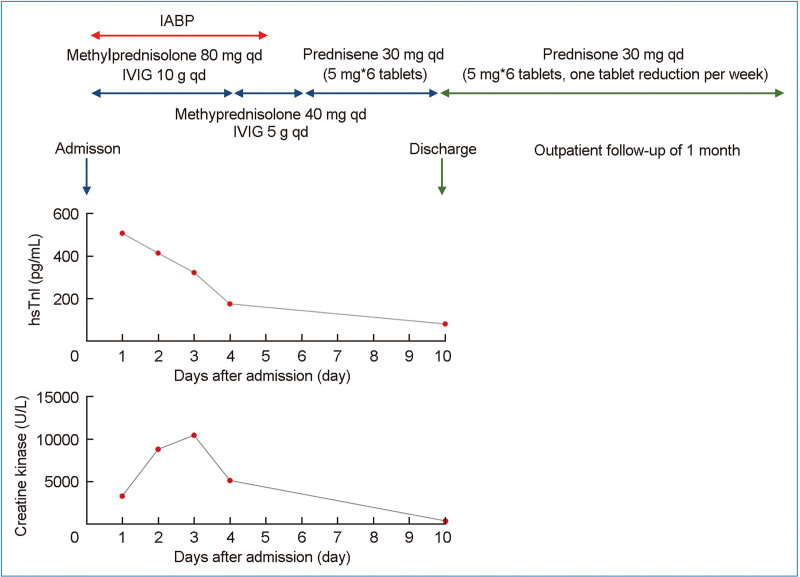
**Treatment plan and the main changes of laboratory examination (troponin, creatine kinase) results after admission**. IABP: intra-aortic balloon pump; IVIG: intravenous immunoglobulin; hsTnl: high sensitive troponin-I.

We considered the possibility of cardiac insufficiency caused by myocarditis. According to expert consensus on fulminant myocarditis in China^[10,11]^, we performed intra-aortic balloon pump (IABP) insertion at the bedside. We also used immunomodulatory therapy with methylprednisolone and intravenous immune globulin (IVIG) as shown in Figure 2. During treatment, the patient’s vital signs gradually stabilized, and her palpitations and chest tightness symptoms improved notably. However, her lower limb pain still intermittently appeared, for which non-steroidal anti-inflammatory drugs were administered for pain relief. While troponin and N-terminal pro B-type natriuretic peptide (NT-proBNP) decreased gradually, creatine kinase (CK) continued to increase, reaching a peak on the fourth day after admission, then gradually decreased and returned to normal on day 10 (Table 1). Cardiac edema and pericardial effusion were also gradually alleviated, and the IABP was removed on day 5.

**Figure 3.**
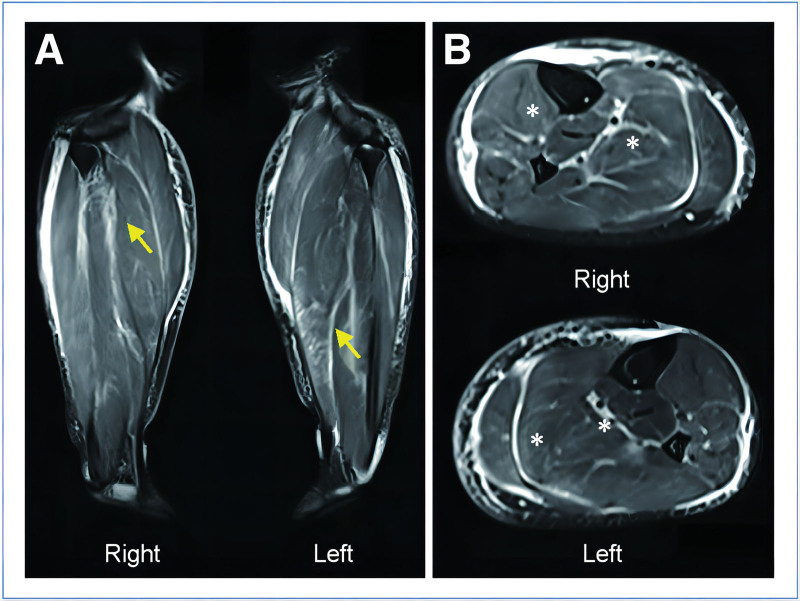
**Magnetic resonance of lower limb muscle. A**, Edema and effusion in muscle space and subcutaneous soft tissue (yellow arrow) from frontal plane of lower limb muscle. **B**, Lower limb muscle magnetic resonance of T2WI sequence showed that there was extensive edema in bilateral calf muscles (asterisk mark) from cross section of lower limb muscle.

**Figure 4.**
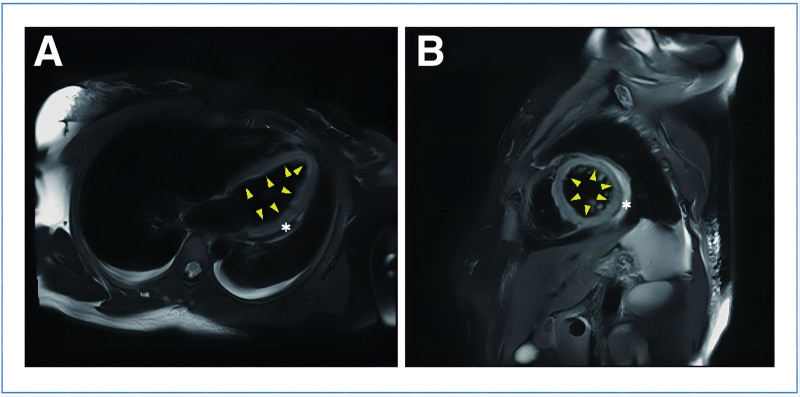
**Cardiac magnetic resonance. A**, Cardiac magnetic resonance of 4-chamber view. **B**, Cardiac magnetic resonance of short axis view.

On day 7 of postadmission, the patient underwent magnetic resonance imaging of the heart and muscles of both lower limbs. The results showed extensive edema in the bilateral calf muscles, and edema and effusion in the muscle space and subcutaneous soft tissues (Figure 3), which was considered to be caused by an inflammatory reaction. Diffuse left ventricular myocardial edema, and a small amount of pericardial effusion were observed (Figure 4). We recommended percutaneous myocardial biopsy and lower limb muscle biopsy, both of which were refused by the patient due to the invasiveness of these procedures. Ten days postadmission, the patient recovered and was discharged. At 1-month outpatient follow-up, her hs-cTnI and CK have returned to normal. It was summarized in Table 2 as a timeline of events that happened during the patient’s hospitalization.

**Table 2:** Timeline of events during the patient’s hospitalization

Time	Events
1 week before admission	Fever of 38°C with cough and expectoration
5 days before admission	The patient developed palpitations, chest tightness, and obvious general fatigue and muscle pain. Also, Troponin, myoglobin, and CK-MB were increased
1 day after admission	Treatment with immunomodulatory therapy (methylprednisolone and IVIG) and IABP
4 days after admission	While troponin and NT-proBNP decreased gradually, CK continued to increase to peak. Reduced doses of immunomodulatory therapy
5 days after admission	Vital signs were stable, cardiac function has recovered and IABP was removed
10 days after admission	CK returned to normal, and the patient had no more symptoms and was discharged

CK-MB: creatine phosphokinase-MB; IABP: intra-aortic balloon pump; IVIG: intravenous immune globulin; NT-proBNP: N-terminal pro B-type natriuretic peptide.

Cardiac magnetic resonance of T2 weighted image (T2WI) sequence showed that there was diffuse left ventricular myocardial edema (yellow arrowhead), a small amount of pericardial effusion (asterisk mark).

## DISCUSSION

During the early stage of the COVID-19 outbreak, SARS-CoV-2 infection was found to cause myocardial damage^[2]^. In 2021, statistics from more than 900 hospitals in the United States showed that the incidence of myocarditis in patients hospitalized with COVID-19 was 16 times higher than in those hospitalized without COVID-19^[12]^. The mechanism of acute myocardial injury caused by COVID-19 may be related to ACE2. ACE2 is widely expressed in both the lungs and the cardiovascular system. Therefore, ACE2-related signaling pathways may also play a role in heart injury. Other possible mechanisms of myocardial injury include a cytokine storm triggered by an imbalanced response by type 1 and type 2 T helper cells^[2,13]^ and respiratory dysfunction and hypoxemia caused by COVID-19, resulting in damage to myocardial cells. In addition to COVID-19 infection, we found that the COVID-19 vaccine can also cause fulminant myocarditis^[14]^.

Skeletal muscle, the largest body tissue involved in glucose metabolism, appears to be among the tissues affected by COVID-19^[5,6]^. Muscle pain is a key symptom that develops during the first 3 days of infection in patients hospitalized with COVID-19 infection^[7–9]^. Muscle tissue also expresses ACE2 receptors^[8]^. Extrapulmonary manifestations of COVID-19 are thought to occur through ACE2 receptor-mediated facilitation of viral entry into the small vessel endothelium of organs such as the bowel, and both smooth and skeletal muscles^[15]^. COVID-19-induced myositis has been speculated to occur through the direct entry of viral particles into the muscles through these cell receptors^[16]^. Most other virus-related muscle inflammation is attributed to T-cell cloning and expansion, and to the production of proinflammatory cytokines, leading to muscle injury^[13]^. It seems reasonable that these mechanisms may also play a role in the pathogenesis of COVID-19. Excessive production of proinflammatory cytokines under hypercatabolic conditions is associated with oxidative stress, which promotes the production of corrosive molecules that cause severe myocyte damage^[17]^.

Laboratory reports have shown remarkable elevation in muscle loss biomarkers, like CK, in up to 27% of hospitalized COVID-19 patients^[18,19]^. The incidence of increasing CK in patients with severe COVID-19 is higher in older males with comorbidities. Higher CK levels have also been associated with increased inflammatory markers^[18]^.

While immunosuppressive drugs are the choice of treatment for most inflammatory myopathies, they can damage the immune system’s ability to resist infectious agents. Therefore, whether to continue or stop immunosuppressive therapy in patients with COVID-19 myositis remains controversial. Glucocorticoids, cyclophosphamide, and IVIG are currently used to treat COVID-19. However, the timing and dosage of immunomodulatory therapy must be carefully balanced because these drugs can worsen COVID-19 complications. New data from cohort studies seem to show the beneficial effects of drugs such as tocilizumab and hydroxychloroquine, which inhibit the inflammatory response through immunomodulation, thus reducing the incidence of cytokine storms^[20,21]^.

In our case, the patient’s lungs were not severely damaged after COVID-19 infection, but her clinical situation was characterized by myocardial damage, heart failure, and muscle damage. Unlike acute myocarditis or fulminant myocarditis, the patient had a significant increase in myoglobin and CK. Although her heart failure improved and troponin level gradually decreased, the patient’s muscle pain was still progressive with increasing CK level. This suggested that severe skeletal muscle injury was caused by COVID-19 infection. Imaging confirmed that the patient’s extent and degree of skeletal muscle injury was significant, while laboratory inflammatory factor tests, including interleukin 1 beta (IL-1β), interleukin 2 (IL-2), and tumor necrosis factor (TNF), were not as severe as expected. Consequently, her doses of immunomodulatory drugs (methylprednisolone and IVIG) were reduced relative to other patients with fulminant myocarditis. The goals of IABP use when myocarditis leads to heart failure are to raise blood pressure, reduce cardiac afterload, improve cardiac function, and help reduce myocardial edema.

## CONCLUSION

COVID-19 can induce myocarditis and myositis, the mechanism of which may involve direct infection of muscles or autoimmune events. For all patients with COVID-19, it is essential to be alert to heart and other muscular damage. Patients with related symptoms should be monitored for troponin, CK, and myoglobin levels, and should be treated in a timely manner to avoid serious complications.

## FUNDING

This work was supported by the National Natural Science Foundation Grants (No. 81800335).

## AUTHOR CONTRIBUTIONS

KM and JL wrote the manuscript and participated in collecting data. FW participated in collecting data. LW performed the imaging examination, CZ also participated in collecting data. DWW revised the manuscript and participated in the writing of the paper.

## CONFLICT OF INTEREST STATEMENT

The authors declare that they have no conflict of interest with regard to the content of this manuscript.

## DATA SHARING STATEMENT

All data generated or analyzed during this study are included in this published article.
